# National Prevalence and Exposure Risk for Cockroach Allergen in U.S. Households

**DOI:** 10.1289/ehp.8561

**Published:** 2005-11-15

**Authors:** Richard D. Cohn, Samuel J. Arbes, Renee Jaramillo, Laura H. Reid, Darryl C. Zeldin

**Affiliations:** 1 Constella Group, LLC, Durham, North Carolina, USA; 2 National Institute of Environmental Health Sciences, National Institutes of Health, Department of Health and Human Services, Research Triangle Park, North Carolina, USA

**Keywords:** allergens, asthma, Bla g 1, cockroach allergen

## Abstract

We characterized the prevalence of cockroach allergen exposure in a nationally representative sample of U.S. homes and assessed risk factors for elevated concentrations.

**Design:**

We used data from the National Survey of Lead and Allergens in Housing, a population-based cross-sectional survey.

**Participants:**

Participants were residents of 831 U.S. homes in the survey.

**Evaluations/Measurements:**

We analyzed allergen, questionnaire, and observational data of 831 U.S. homes.

**Results:**

Cockroach allergen (Bla g 1) concentrations exceed 2.0 U/g, a level associated with allergic sensitization, in 11% of U.S. living room floors and 13% of kitchen floors. Concentrations exceed 8.0 U/g, a level associated with asthma morbidity, in 3% of living room floors and 10% of kitchen floors. Elevated concentrations were observed in high-rise apartments, urban settings, pre-1940 constructions, and households with incomes < $20,000. Odds of having concentrations > 8.0 U/g were greatest when roach problems were reported or observed and increased with the number of cockroaches observed and with indications of recent cockroach activity.

**Conclusions:**

Household cockroach allergen exposure is characterized in a nationally representative context. The allergen is prevalent in many settings, at levels that may contribute to allergic sensitization and asthma morbidity.

**Relevance to Clinical or Professional Practice:**

Likelihood of exposure can be assessed by consideration of demographic and household determinants.

Asthma, a chronic respiratory disease characterized by episodes of airway inflammation and narrowing, represents a significant public health problem. The prevalence of asthma in the United States has increased considerably since 1980 (Mannino et al. 1998), coinciding with an increasing tendency among Americans to spend time indoors [U.S. Environmental Protection Agency (EPA) 1996]. These patterns have led researchers to carefully examine exposure to indoor allergens as an important risk factor for asthma.

It has been clearly established that exposure to cockroach allergen is one such risk factor. Many studies have demonstrated this association, including some that have found that levels of cockroach allergen in homes are one of the strongest risk factors predictive of allergic sensitization and asthma morbidity in children (Arruda et al. 2001; Call et al. 1992; Chapman et al. 1996; Crain et al. 2002; Eggleston et al. 1998; Gelber et al. 1993; Rosenstreich et al. 1997; Sarpong et al. 1997). It has been estimated that 26.1% of the U.S. population exhibits allergic sensitization to the German cockroach, based on rates of positive skin tests from the Third National Health and Nutrition Examination Survey (NHANES III) (Arbes et al. 2005a). However, estimates of exposure in a nationally representative sample of homes have never been reported.

Previous studies examining levels of cockroach allergens and associated characteristics in U.S. homes have targeted specific populations such as single metropolitan areas and inner-city neighborhoods (Chew et al. 1998; Gehring et al. 2004; Kitch et al. 2000; Leaderer et al. 2002). Such studies are critical for identifying important relationships in high-risk populations but do not permit a more general understanding of allergen exposure. For example, a major study of the homes of asthmatic children demonstrated key exposure–disease relationships but involved a nonrandom sample that exhibited wide variation in cockroach allergen levels between various metropolitan areas (Huss et al. 2001). Nationally representative data are needed to provide a broadly applicable characterization of household cockroach allergen levels and their determinants. For this article, we took such data from the National Survey of Lead and Allergens in Housing (NSLAH), conducted from 1998 through 1999 by the National Institute of Environmental Health Sciences (NIEHS) and the U.S. Department of Housing and Urban Development (Jacobs et al. 2002; Vojta et al. 2002).

The weighted NSLAH population is, by design, comparable with the U.S. population of eligible housing units: 28% in urban areas with population > 1 million, 39% with children < 18 years of age, 80% white, 8% Hispanic, and 80% above the poverty level.

The objectives of this research are to provide the first nationally representative estimates of cockroach allergen prevalence within households and to identify demographic factors and housing characteristics associated with high cockroach allergen levels. Achieving these objectives will provide a characterization of household cockroach allergen exposure on a nationwide basis, assist clinicians in assessing the likelihood of a patient’s exposure, and influence research hypotheses for intervention studies.

## Materials and Methods

The NSLAH was a cross-sectional survey of the U.S. population of 96 million permanently occupied, noninstitutional housing units that permit resident children, and was carried out in 1998–1999. A complex, multistage design was used to sample and gain participation from 831 housing units containing 2,456 individuals. The staged design involved the selection of 75 primary sampling units (defined as metropolitan statistical areas or counties) across the United States, followed by the sampling of segments (defined as contiguous blocks) within each primary sampling unit, and then the sampling of housing units within each segment. Among 1,984 housing units initially selected to be recruited into the study, 980 were determined to be eligible during screening, 229 were found to be ineligible, and 775 did not complete sufficient screening to determine eligibility. Assuming that the eligibility rate among these 775 is the same as the rate among households of known eligibility, the surveyed population of 831 housing units constitutes a response rate of 52% of an estimated 1,608 eligible units. Demographic comparisons with both the American Housing Survey (U.S. Census Bureau 2005) and the Current Population Survey (U.S. Census Bureau and Bureau of Labor Statistics 2001) suggest that the participating housing units are highly representative of the intended target population. At each home, a questionnaire was administered to an adult householder, environmental samples were collected, and observations were recorded. A detailed description of the survey design, methodology, and response rates can be found elsewhere (Jacobs et al. 2002; Vojta et al. 2002). The survey was approved by the NIEHS Institutional Review Board on 16 June 1998, and, in each home, informed consent was obtained in writing from an adult household member.

### Sample collection.

Dust samples used in this analysis were collected from the kitchen floor, living room floor, upholstered living room furniture, a randomly selected bedroom bed, and bedroom floor, using a Eureka Mighty-Mite 7.0-A vacuum cleaner (Eureka Company, Bloomington, IL). A 19 mm × 90 mm cellulose extraction thimble (Whatman International, Ltd., Maidstone, UK) was placed in the distal end of the vacuum’s extension tube, sealed with a rubber O-ring, and covered with a clean crevice tool. Defined floor areas or perimeters, upholstery surfaces, and bedding layers were vacuumed over specified time intervals. Details of dust collection protocols are described elsewhere (Vojta et al. 2002).

At the laboratory, dust samples were sieved through 425-μ m pore grating, weighed, and divided into 100-mg aliquots of fine dust. Dust aliquots were extracted in borate-buffered saline and clarified by centrifugation. Supernatants were decanted and stored at –20°C. Cockroach allergen was measured by a two-site, monoclonal antibody enzyme-linked immunosorbent assay (Pollart et al. 1991). Allergen concentrations reported in this article are of Bla g 1, measured in units per gram dust. The lower limit of detection of the assay varied and was 0.10 U/g for 94% of assayed samples, 0.20 U/g for 4%, and 0.40 U/g or 0.80 U/g for most others. Allergen loads are calculated as the product of concentration and dust weight, measured in units of allergen per square meter of sampled area. Because some dust samples were not collected and because some samples had too little dust to analyze for all allergens, there were some missing concentration values. However, cockroach allergen measurements were available from at least one room for 826 (99.4%) of the homes entered into the study.

### Statistical analyses.

We selected factors for analysis on the basis of hypothesized relevance gleaned from the literature or other sources. All percentages, correlations, means, percentiles, and odds ratios (ORs) were weighted to represent the U.S. population of permanently occupied, noninstitutional housing units that permit resident children. The statistical weighting included the application of a nonresponse adjustment factor to the weights of the surveyed housing units, to ensure that they also represent the eligible housing units that did not participate in the survey. A detailed description of the statistical weighting for the NSLAH can be found elsewhere (Vojta et al. 2002). We calculated Spearman rank correlation coefficients as a robust measure of association between allergen concentrations. ORs and 95% confidence intervals (CIs) were estimated using logistic regression and Wald *F*-test statistics. We developed standard errors (SE), CIs, and *p*-values in accordance with the complex survey design using Taylor series linearization methods. Statistical analyses were conducted using SAS software (release 8.2; SAS Institute Inc., Cary, NC) and SUDAAN (release 8.0; Research Triangle Institute, Research Triangle Park, NC).

## Results

### Distributions of allergen concentrations.

Table 1 shows the distribution of cockroach allergen concentration and load in U.S. homes. Detectable concentrations of cockroach allergen were found in at least one sampling location of an estimated 63% of U.S. homes. Kitchen floors exhibited the highest levels, with 13% having concentrations > 2.0 U/g and nearly 10% exceeding 8.0 U/g, levels previously found to be associated with significantly increased allergic sensitization and asthma morbidity rates, respectively (Eggleston et al. 1998; Rosenstreich et al. 1997). Living room floors exhibited the greatest prevalence of allergen, with 44% above the lower limit of detection.

Among samples containing detectable cockroach allergen from locations with recorded collection areas, the bedroom floor exhibited the highest median load (0.251 U/m^2^) and the highest median weight of sampled dust (459 mg). Samples of comparable median dust weight were collected from the living room floor (290 mg) and the bedroom bed (287 mg); thus, the higher median cockroach allergen load seen in living room floors is mostly a function of higher concentrations in that location.

Spearman rank correlations of cockroach allergen concentrations between like surfaces and same-room sampling locations ranged from 0.17 to 0.57. We investigated relationships between these correlations and the cleaning methods recently used in the home (Table 2). Generally, higher correlations were observed in areas that were swept (0.46–0.77) or mopped (0.38–0.93) compared with vacuumed (0.10–0.57) when last cleaned. We also conducted stratified analyses controlling for type of flooring to assess any evidence of confounding with cleaning method and obtained similar patterns in the correlations among cleaning methods.

Among other measured allergen concentrations, cockroach was most highly correlated with mouse (Mus m 1; range, 0.14–0.25) across the sampling locations and endotoxin (range, 0.09–0.30).

### Cockroach allergen and demographics.

To investigate associations between cockroach allergen and various demographic factors, we compared the distributions of concentration across levels of the factors shown in Table 3. Living room floor concentrations are reported because that location exhibited the greatest prevalence of detectable allergen and offers significant opportunity for exposure. Although higher concentrations were measured in the kitchen, more residents reported spending significant time in the living room (79% of residents spent > 1 hr/day and 33% spent > 4 hr/day in the living room, compared with 58% and 7% for the kitchen, respectively). Although living room floor concentrations are illustrated here, similar patterns and trends were obtained for other locations; any exceptions are noted below.

The highest prevalence of elevated concentrations was observed in high-rise apartments. Generally, higher concentrations were also observed in homes built before 1940, urban areas, low-income households, and multifamily structures. Some differences were also seen according to geographic region of the United States, with somewhat higher living room floor levels in the Northeast and Midwest. However, results obtained using dust sampled from the kitchen floor were reversed from those in Table 3, with higher levels in the South and West (12.9% and 11.5% exceeding 8.0 U/g, respectively) compared with the Northeast and Midwest (4.3% and 6.6%, respectively). Regional differences and associations with construction year were less pronounced in bedroom concentrations.

Detectable concentrations were generally prevalent in all demographic categories; the lowest rate of detection was 29.9% among western households.

With regard to income in particular, patterns seen in Table 3 were generally preserved in separate analyses of household income subgroups of more than and less than $20,000. One exception involved type of dwelling within the < $20,000 population. Among these low-income households, living room floor concentrations exceeded the 2.0-U/g threshold most often in high-rise apartments (42.5%), similar to the full-population results. However, exceedance rates were higher among low-income duplex/triplex homes (29.0%), detached single-family homes (17.7%), and mobile homes (15.4%), relative to low-rise apartments (15.7%) and row houses (11.3%), compared with the full-population results.

Table 4 further describes regional aspects of living room floor cockroach allergen exceeding 2.0 U/g. Some categories are combined relative to Table 3, and the 2.0-U/g threshold is illustrated to preserve adequate sample sizes in each category and region. Data in Table 4 illustrate general consistency among regions with respect to overall trends; each region exhibits somewhat higher concentrations in older constructions, lower income homes, urban homes, and multifamily buildings. Within every demographic category, midwestern households consistently show the highest prevalence of concentrations exceeding 2.0 U/g compared with the other regions.

In contrast and as mentioned above, generally higher kitchen floor cockroach allergen concentrations were observed in the South and West. Among southern homes with household incomes < $20,000, 31.9% of kitchen floors exceeded 2.0 U/g and 24.2% exceeded 8.0 U/g. Results for low-income western homes were similar (30.8% and 28.5%, respectively). All such results were lower in low-income northeastern (17.2% and 7.6%) and midwestern (18.1% and 18.1%) homes.

### Household characteristics and exposure risk.

After accounting for demographic factors, specific household characteristics were analyzed to investigate their ability to predict higher levels of living room floor cockroach allergen relative to two thresholds: 2.0 U/g and 8.0 U/g. Results are displayed in Table 5. As expected, indicators of cockroach activity were very strong predictors of elevated cockroach allergen levels. We investigated allergen levels relative to the time frame and degree of infestation and found that ORs for elevated concentrations consistently increased with more recent observation of cockroaches and with numbers of cockroaches observed. Floor sweeping or mopping was also associated with higher levels compared with vacuuming, but not to a degree that achieved statistical significance in a subset of the population. (To support a valid comparison of cleaning methods, this analysis was restricted to homes for which some hard flooring was observed in the living room. Mats or area rugs were also observed in some of these rooms, and these were not excluded.) Associations were found between allergen levels > 8.0 U/g and various factors, including food debris or moisture observed in the room and the presence of a smoker in the home.

Cockroach allergen in dust sampled from the kitchen floor yielded results generally comparable with those displayed in Table 5, in terms of both the directionality and relative magnitude of ORs among the factors. The only difference with regard to the overall pattern involves the two thresholds of 2.0 U/g and 8.0 U/g; ORs for the kitchen floor tended to be similar at the two thresholds and to fall somewhere between the more extreme living room floor ORs. For example, residents reporting problems with cockroaches exhibited ORs of 15.5 and 14.0 for kitchen floor allergen concentrations exceeding 2.0 U/g and 8.0 U/g, respectively. The only individual factor exhibiting a substantive difference between the living room floor and kitchen floor results involved field staff observation of cockroach stains; this factor yielded ORs of 26.7 (95% CI, 11.0–65.0) and 27.3 (95% CI, 11.1–67.0) for kitchen floor concentrations exceeding 2.0 U/g and 8.0 U/g, respectively.

Restating our results in terms of positive and negative predictive value, 86% of residents reporting problems with cockroaches did in fact have detectable concentrations at one or more sites, with 58% > 2.0 U/g and 38% > 8.0 U/g. Only 16% of residents indicating no such problems had detectable levels > 2.0 U/g, and 4% > 8.0 U/g. Although 100% of residents who reported seeing > 50 cockroaches/day had detectable levels > 8.0 U/g, resident reporting of between 5 and 50 cockroaches per day was also a strong predictor of levels > 2.0 U/g and 8.0 U/g (96.6% and 63.0%, respectively). Other useful predictors of exceeding these thresholds included resident reporting of cockroaches seen within the past week (77.9% and 58.7%) or month (74.3% and 53.1%) and field staff reports of roach stains or live or dead roaches in at least one location in the home (71.2% and 52.5%).

## Discussion

In this article we provide the first nationally representative estimates of household cockroach allergen prevalence and find that 11% of U.S. living room floors and 13% of kitchen floors exhibit elevated concentrations relative to a 2.0-U/g threshold previously established as related to allergic sensitization (Eggleston et al. 1998). We also find detectable levels in at least one location of an estimated 63% of all homes.

In the National Cooperative Inner-City Asthma Study, children with sensitivity to cockroach allergen who were exposed to bedroom levels > 8.0 U/g had asthma hospitalization rates that were 3.7 times higher than sensitive children with lower levels of exposure (Rosenstreich et al. 1997). On a national scale, we have found that 3% of U.S. living room and bedroom floors and 10% of kitchen floors exhibit elevated concentrations relative to this 8.0-U/g threshold.

Elevated cockroach allergen levels are most prevalent in high-rise apartments, urban settings, pre-1940 constructions, and households with incomes < $20,000. However, the allergen is not restricted to low-income environments; levels > 2.0 U/g were detected on the living room floors of 7% of households with annual incomes > $60,000.

Although there are some regional differences in magnitude, the same demographic factors are generally associated with elevated cockroach allergen levels in each geographic region. Demographic distributions themselves provide one possible explanation for the greater prevalence of elevated living room floor cockroach allergen in the Northeast compared with the South, because the Northeast target population includes relatively more pre-1946 constructions (43% vs. 14%) and more households in large urban areas (34% vs. 23%).

After accounting for the demographics, elevated exposure risk is most strongly associated with reported or observed cockroach activity and increases consistently with more recent or more prevalent activity. This is particularly informative in the context of cockroach allergen abatement studies that have focused on the impact of cleaning and insecticide application (Arbes et al. 2003, 2004; Eggleston and Arruda 2001; Eggleston et al. 1999; Gergen et al. 1999). Our results are consistent with the potential success of such a strategy. They also suggest that resident reports on cockroach activity alone may have some reliability as one indicator of such success, apart from in-house measurements or field staff observation.

Cockroach allergens are derived from several sources, including secretions, excretions, dead bodies, and associated debris. Airborne cockroach allergens are associated primarily with larger particles than are animal allergens, and after disturbance they fall and settle rapidly (Eggleston and Arruda 2001). The generally low room-to-room correlations observed in this study suggest limited airborne transmission of the allergen, consistent with these physical properties. The results also suggest that the degree of correlation is associated with the method of cleaning; weaker correlations were observed among vacuumed surfaces compared with those that are mopped or swept. The practices of mopping or sweeping may tend to cause more spreading of the allergen and therefore higher correlation. Such causality is not conclusive, however. It is also possible that the observed correlation is a direct computational result of the generally higher concentrations observed in homes in which mopping and sweeping are employed. Residents reported that floors had been cleaned in most participating households within 2 weeks before dust sampling (89% of living room floors and 92% of kitchen floors).

The major strength of this study is its national representativeness. The weighted characteristics of the surveyed homes compared favorably with those of other national housing surveys (Vojta et al. 2002). Our results illustrate the importance of achieving this nationally representative sample in characterizing exposure. Researchers who examined an intentionally nonrepresentative sample of homes in eight North American metropolitan areas reported detecting Bla g 1 (based on a lower limit of 0.4 U/g) in 9.4% of homes on average (range, 1.5–21.5%), using a combined dust sample collected from multiple household sites (Huss et al. 2001). In contrast, based upon a weighted average of concentrations from all household sites and a nationally representative sample, we found detectable concentrations > 0.4 U/g in 27.4% of U.S. homes.

The major limitation of the study is its cross-sectional design. Dust samples were collected at a single point in time because repeated visits to the home were not feasible. However, this was the most efficient method for achieving the stated objective of estimating and characterizing indoor allergen levels on a national scale. Sampling was conducted across seasons so as to mitigate any possible seasonal bias.

This study surveyed homes for the Bla g 1 allergen from the German cockroach *Blattella germanica*, a small organism responsible for the primary U.S. exposure. The estimated prevalence of detectable Bla g 1 concentrations in 63% of homes is consistent with other research that found 52% prevalence in a regional sample of child care facilities (Arbes et al. 2005b). Nevertheless, our absolute estimates of the prevalence of detectable concentrations may be affected by greater assay variability at very low concentrations and should be interpreted with some caution. They are reported in Table 3 primarily to support comparative analyses and are shown together with the likely more stable results at the 2-U/g and 8-U/g thresholds. Our results relative to these thresholds are also very consistent with the aforementioned child care study, which found that 10.4% of daycare floor Bla g 1 concentrations exceeded 2 U/g and 2.3% exceeded 8 U/g (Arbes S, personal communication).

This study characterizes the prevalence of cockroach allergen in U.S. homes and illustrates factors influencing the risk of exposure to elevated concentrations. These results may help clinicians to assess whether a patient is likely to be exposed and suggest measures to reduce this exposure. Our results also lend important context to the potential impact of abatement studies of cockroach allergen.

## Figures and Tables

**Table 1:** Estimated distribution of cockroach allergen concentration and load in U.S. homes.

		Concentration				
Sampling location	No. of homes sampled	Percent detectable (SE)	Percent > 2.0 [U/g (SE)]	Percent > 8.0 [U/g (SE)]	Median^a^ detectable concentration (U/g)	Median^a^ detectable load (U/m^2^)
Bedroom bed	767	6.1 (0.8)	1.3 (0.4)	0.5 (0.2)	0.292	0.037
Bedroom floor	762	17.6 (1.6)	6.7 (0.8)	3.2 (0.7)	0.769	0.251
Kitchen floor^b^	764	28.5 (1.9)	13.4 (1.3)	9.5 (1.0)	1.376	—
Living room floor	763	44.4 (2.1)	10.7 (1.5)	2.7 (0.7)	0.927	0.152
Living room upholstery^b^	729	38.4 (2.0)	8.8 (1.2)	1.1 (0.4)	0.779	—

a Median among all households is less than the lower limit of detection; therefore, median among detectable levels is reported.

b Vacuumed area not recorded; unit load not calculated.

**Table 2:** Spearman rank correlations among cockroach allergen concentrations at different sampling locations in U.S. homes.

		Population, cleaning method^a^		
Sampling location pair	Full population	Floors swept	Floors mopped	Floors vacuumed
Bedroom bed, bedroom floor	0.25	0.46	0.44	0.16
Bedroom bed, living room upholstery	0.17	0.62	0.93	0.10
Bedroom floor, kitchen floor	0.42	0.66	0.64	0.26
Bedroom floor, living room floor	0.28	0.56	0.74	0.25
Kitchen floor, living room floor	0.28	0.61	0.38	0.24
Living room floor, living room upholstery	0.57	0.77	0.53	0.57

a Subpopulations defined by use of the same cleaning method for floors in proximity to both sampling locations in the pair. Sample size ranges were 678–732 (all homes), 37–79 (swept), 18–50 (mopped), and 65–560 (vacuumed).

**Table 3:** Estimated percentage of U.S. households with detectable living room floor cockroach allergen, with concentrations exceeding 2.0 U/g and 8.0 U/g, and OR for concentrations above 8.0 U/g, according to demographic factors.

Factor	No. of homes sampled	Percent detectable (SE)	Percent > 2.0 U/g (SE)	Percent > 8.0 U/g (SE)	OR for Bla g 1 > 8.0 U/g (95% CI)
Type of dwelling (*p* = 0.010)^a^					
Detached single family	502	44.6 (2.9)	9.7 (1.8)	0.8 (0.4)	Reference
Duplex/triplex	56	48.3 (8.4)	15.7 (5.5)	4.8 (3.2)	5.97 (1.32–26.9)
Row house	39	42.6 (9.6)	12.6 (4.0)	5.9 (1.7)	7.39 (2.64–20.7)
Low-rise apartment (1–4 floors)	76	41.1 (7.5)	12.3 (3.8)	6.1 (2.9)	7.56 (2.01–28.5)
High-rise apartment (≥ 5 floors)	15	84.3 (7.3)	45.7 (13.2)	37.3 (13.6)	70.0 (16.6–295.9)
Mobile home	36	37.0 (8.7)	7.5 (5.6)	5.3 (5.2)	6.56 (0.60–71.7)
Construction year (*p* = 0.001)					
1978–1998	198	37.7 (4.1)	8.8 (2.6)	2.1 (1.2)	Reference
1960–1977	245	40.8 (3.9)	7.5 (1.9)	1.6 (0.7)	0.75 (0.17–3.29)
1946–1959	131	50.4 (4.9)	12.8 (3.2)	1.4 (0.8)	0.65 (0.12–3.50)
1940–1945	43	55.0 (6.9)	4.7 (2.9)	2.6 (2.1)	1.22 (0.17–8.74)
1939 or earlier	146	52.1 (4.0)	19.2 (3.7)	6.7 (2.4)	3.29 (0.87–12.4)
Geographic region (*p* = 0.002)					
Northeast	137	46.7 (5.1)	12.1 (3.0)	3.2 (1.5)	1.78 (0.42–7.52)
Midwest	188	58.4 (3.4)	15.3 (3.4)	3.9 (1.6)	2.21 (0.57–8.52)
South	253	41.7 (3.9)	8.9 (2.9)	1.8 (0.9)	Reference
West	185	29.9 (4.5)	7.2 (1.7)	2.6 (1.3)	1.48 (0.35–6.27)
Urbanization (*p* = 0.014)					
MSA ≥ 1 million population	255	53.1 (4.4)	17.5 (3.2)	4.7 (1.6)	3.15 (1.06–9.37)
MSA < 1 million population	379	40.2 (2.4)	6.7 (1.9)	1.5 (0.6)	Reference
Non-MSA	129	42.2 (4.7)	10.6 (3.0)	2.8 (1.6)	1.83 (0.43–7.83)
Household income (*p* = 0.003)					
$0–19,999	172	53.5 (4.4)	18.5 (3.5)	8.3 (2.3)	12.1 (2.05–71.7)
$20,000–39,999	214	47.4 (4.7)	11.5 (2.4)	1.9 (1.0)	2.53 (0.37–17.6)
$40,000–59,999	139	34.9 (5.1)	3.4 (1.4)	0.0 (0.0)	—b
≥ $60,000	181	41.9 (4.0)	7.3 (2.4)	0.7 (0.6)	Reference
No. of units in building (*p* = 0.062)					
Single family^c^	651	43.2 (2.2)	9.8 (1.6)	1.9 (0.5)	Reference
Multifamily	112	51.8 (5.9)	17.2 (4.2)	8.5 (3.1)	4.89 (1.87–12.8)

MSA, metropolitan statistical area.

a *p*-Values for Wald *F*-test for equality of geometric mean concentrations among all levels of the factor.

b No observations to support OR calculation.

c Single-family housing is defined as having fewer than five units in the building.

**Table 4:** Estimated percentage (SE) of U.S. households with living room floor cockroach allergen concentrations exceeding 2.0 U/g, according to demographic factors and region.

	Region			
Factor	Northeast	Midwest	South	West
Construction year				
1978–1998	8.8 (6.7)	13.7 (5.6)	8.1 (4.1)	6.9 (4.1)
1960–1977	6.0 (4.4)	10.7 (4.0)	7.5 (3.6)	5.1 (2.0)
1946–1959	8.6 (5.7)	20.0 (7.2)	13.8 (5.2)	2.9 (2.9)
1945 or earlier	17.2 (5.0)	17.7 (5.1)	9.4 (7.2)	15.6 (7.3)
Urbanization				
MSA ≥ 1 million population	18.7 (7.9)	22.1 (8.7)	19.2 (6.4)	11.8 (3.0)
MSA < 1 million and non-MSA	8.2 (1.5)	13.7 (3.7)	5.7 (3.1)	4.1 (2.0)
Household income				
$0–19,999	22.6 (11.0)	25.6 (6.2)	15.7 (5.9)	12.9 (7.4)
$20,000–39,999	10.1 (4.8)	17.0 (5.2)	10.5 (5.0)	7.4 (1.1)
≥ $40,000	4.4 (2.3)	8.0 (3.7)	4.6 (2.5)	5.0 (2.7)
No. of units in building				
Single family^a^	11.3 (3.8)	14.3 (3.5)	8.6 (2.9)	4.7 (1.8)
Multifamily	15.5 (8.9)	25.3 (9.9)	12.0 (6.8)	19.0 (7.4)

MSA, metropolitan statistical area.

a Single-family housing is defined as having fewer than five units in the building.

**Table 5:** Adjusted^a^ ORs (95% CIs) for increased living room floor cockroach allergen concentration, according to household characteristics

	OR for higher Bla g 1 when characteristic is present	
Characteristic	Bla g 1 > 2.0 U/g	Bla g 1 > 8.0 U/g
Reported problems with cockroaches^b^	4.62 (2.67–7.96)	65.47 (16.93–253.2)
Roaches last seen 2–4 months ago^b^	2.4 (0.6–10.0)	11.8 (1.2–118.7) c
Roaches last seen within the last month^b^	6.9 (3.8–12.6)	114.7 (28.0–469.2)
Roaches last seen within the last week^b^	8.3 (4.2–16.3)	222.9 (46.9–1,060)
Average of < 5 roaches seen per day^b^	5.5 (2.8–10.7)	78.2 (14.7–415.5)
Average of 5–50 roaches seen per day^b^	8.9 (2.4–32.9)	208.2 (16.3–2,664)
> 50 roaches seen per day on average^b^	153.4 (13.6–1,731)	599.0 (95.6–3,753) c
Live/dead cockroaches in room^d^	11.48 (3.44–38.24)	155.51 (25.38–953.0)
Cockroach stains in room^d^	2.94 (0.84–10.31)	9.29 (1.42–60.91)
Reported problems with rodents	1.11 (0.51–2.41)	1.31 (0.39–4.39)
Rodents in room^d^	1.16 (0.21–6.39)	3.97 (0.44–36.19)
Noncarpeted (vs. wall-to-wall carpeted) floor^d^	1.06 (0.45–2.48)	3.55 (0.83–15.19)
Floor mopped or swept (vs. vacuumed) when last cleaned^e^	3.43 (0.74–15.82)	1.59 (0.30–8.53)
Food debris in room^d^	1.04 (0.53–2.04)	4.70 (1.93–11.45)
Moisture in room^d^	0.68 (0.23–2.08)	5.60 (1.29–24.33)
Room humidity > 60%^d^	2.33 (1.36–3.98)	1.57 (0.51–4.85)
Air conditioning not used in home	1.72 (0.93–3.18)	3.20 (0.89–11.56)
Smoker(s) in home	1.41 (0.88–2.28)	3.77 (1.16–12.32)

a Independently modeled to adjust for type of dwelling, construction year, and household income. Each variable was defined as in Table 2 except for income, which was dichotomized at $30,000 to preserve adequate sample size for each factor combination. Sample sizes in this table range between 446 and 686 households having data available on both the characteristic and the allergen, unless otherwise noted.

b These ORs are relative to households with no reported problems with cockroaches.

c Crude OR; adjustment cannot be calculated from the distribution for this factor and level.

^d^Based on in-home observation by field staff.

^e^Adjusted ORs for subpopulation of 148 homes with some hard flooring observed and having all data available.
